# Insecticide substitutes for DDT to control mosquitoes may be causes of several diseases

**DOI:** 10.1007/s11356-012-1145-0

**Published:** 2012-09-06

**Authors:** Md. Mahbubar Rahman

**Affiliations:** Department of Entomology, Bangabandhu Sheikh Mujibur Rahman Agricultural University (BSMRAU), Gazipur, Bangladesh

**Keywords:** Persistent organic pollutant, DDT, Mosquito coil, Aerosol, Integrated vector management

## Abstract

Malaria continues to be a public health problem in Bangladesh, despite efforts in the 1960s to eradicate the vectors through the use of DDT. At one point, eradication of malaria was acclaimed but later on it reappeared. The use of DDT is no more legally allowed in Bangladesh, which has been officially replaced by a number organophosphates and/or synthetic pyrethroids and their combinations in addition to the integrated vector management (IVM) package. IVM being a community approach is still to go a long way to be mass popular. Adulticides, larvicides, residual sprays, mosquito coil, insecticide-impregnated curtain, aerosol, etc. still serve as the major weapons of mosquito control. Thus, mosquito control still mostly depends on chemical insecticides. Although the use of DDT is banned in Bangladesh, there are reports on its illegal use in different forms. Moreover, there is tons of leftover DDT in Bangladesh, which is likely to cause several diseases. As per one report, about 500 MTs of DDT stockpiles are lying in the Medical Sub-Depots at Chittagong for over a period of 26 years. DDT is a persistent organic pollutant pesticide, which can cause diseases like cancer, endocrine disorder, disruption of immune system, embryonic abnormality, reproductive disorder, etc. Other chemical insecticides, which are replacing DDT, are also not free of hazardous impacts. IVM thus appears to be a wise approach requiring concerted efforts for the management of mosquito to control malaria. Such an IVM comprises use of *Bacillus thuringiensis* Berliner var. *israelensis*, methoprene, biocontrol agents, cleaning of breeding sites, pyrethroid-impregnated curtain, etc. Therefore, a wise effort should be adopted to completely stop the use of DDT, eliminate its stockpiles wherever they are in Bangladesh and to popularise the IVM, not the chemicals-based alternatives throughout the country.

## Introduction

Malaria caused by *Plasmodium vivax*, *Plasmodium ovale*, *Plasmodium malariae* and *Plasmodium falciparum* still is a serious threat in many parts of Bangladesh (Banglapedia 2006). These are transmitted by seven species of mosquitoes. Among them, *Anopheles dirus*, *Anopheles minimus*, *Anopheles philippinensis* and *Anopheles sundaicus* are the most important species. *A. dirus* is a wild species found both outdoors and indoors, in hilly forested and foothill areas. *A. minimus*, a primary vector occurring in hilly forested and foothill areas was eliminated through Malaria Eradication Program (MEP) using DDT but has recently reappeared. *A. philippinensis* occurring in flood plain deltaic region also eliminated through MEP is scarcely found now, and *A. sundaicus* occurring in coastal areas was virtually eliminated through MEP but has recently reappeared. *Anopheles annularis* usually found in large numbers is an important vector in some places. *Anopheles aconitus* although not common is a secondary vector in some places, and *Anopheles vagus* fairly common all over Bangladesh has been recently incriminated as vector. Besides, 79 culicine species have been recorded including *Culex quinquefasciatus*, which is a vector of filariasis, and *Aedes aegypti* and *Aedes albopictus* are suspected as vectors of dengue. DDT because of its health hazardous effects has been banned in 1998 in Bangladesh in compliance with the Stockholm Convention (Rahman 2007), and a number of chemicals in different forms are now used as substitutes for DDT. Now the question is: Are these chemical substitutes free of any health hazardous effects? This article provides answers to this question.

## Materials and methods

Available literature has been reviewed to answer the above-mentioned question. A visit to the four Medical Sub-Depots (MSDs) at Agrabad, Chittagong to physically verify the recent status of the DDT stockpiles has been undertaken. The author recently carried out a sample market survey and collected information on the substitutes for DDT available and sold in Bangladesh. The information thus collected and reviewed has been compiled into this article.

## Results and discussion

### Malaria situation in Bangladesh

**Fig. 1:**
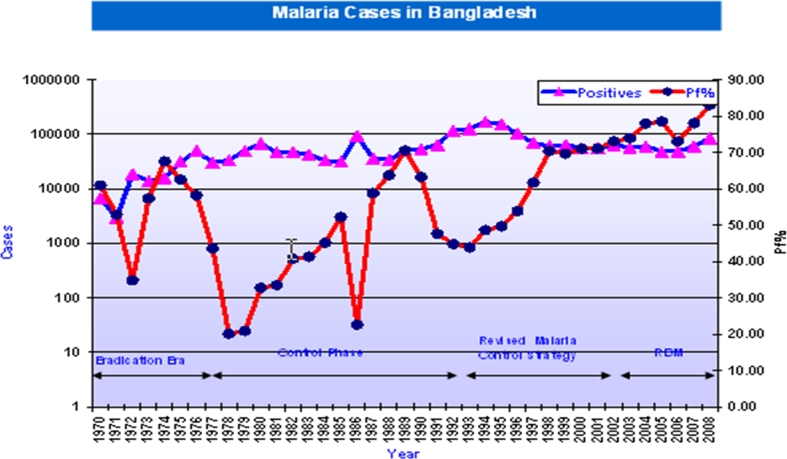
Confirmed malaria cases during 1970–2008 (WHO 2009)

MEP was successfully carried out with residual spray of DDT supplied by WHO during 1960–1977 (eradication era) resulting in near eradication of malaria/mosquito in 1977 (Fig. 1). The use of DDT was continued for mosquito control during 1971–1992 (control phase) with DDT produced inside the country along with donor-supplied DDT. Subsequently, the Revised Malaria Control Strategy (1993–2002) was adopted (WHO 2009) that included restricted use of DDT and other control measures including insecticide-treated nets (ITNs) and long lasting insecticidal nets (LLINs). The DDT was banned in 1998; following which, the use of DDT was highly restricted. The malaria again started reappearing. The resurgence of malaria continued (Roll Back Malaria). The current programme is promoting LLINs and ITNs amongst the community as a vector control measure in the epidemic-prone border areas in 34 upazillas which has increased tremendously in last few years. A total of 2.90 million bed nets (LLINS + ITNs) were distributed and 5.79 million people are covered by it. However, its coverage in high-endemic districts ranges from 40 to 63 % (WHO 2010). Approximately 33.6 % of the total population is at risk of malaria. Majority of malaria cases are reported from 13 out of the total 64 districts in the country. About four million populations living in 34 upazillas of eight of the 13 districts live in the epidemic-prone border areas. Focal outbreaks occur every year, and the response to control the epidemic is inadequate. Malaria cases are grossly underreported due to shortcomings in surveillance and information.

### DDT stockpiles

DDT is no more produced in the country and is also not imported. The discontinuation of DDT as well as import of substandard DDT caused obsolete DDT stockpiles in different locations of Bangladesh. The DDT stockpiles of 602.389 MTs comprise 482.904 MTs of substandard DDT at a single location in four MSDs in Chittagong, 101.69 MTs of DDT technical at Bangladesh Chemical Industries Corporation, Chittagong, 12.795 MTs of DDT 75 WP at district godowns of Directorate of Health and 0.005 MTs of DDT 75 WP at district godowns of Department of Agricultural Extension (Rahman 2007). The very poor storage conditions of the MSDs are resulting in seepage, pilferage, weathering and misuse of DDT, which are contaminating the environment. These are suspected, as reported elsewhere (ATSDR 2002), to cause serious health hazards such as cancers and tumours (particularly breast cancer in women), neurobehavioural impairment including learning disorders, endocrine system disruption, reproductive deficits and sex-linked disorders (birth defects/premature birth of baby), a shortened period of lactation in nursing mothers and increased rate of diabetes to the surrounding affected human residents.

### Substitutes for DDT for mosquito control and their adverse effects

Currently, peoples are using different insecticidal substitutes for DDT in getting rid of mosquito biting, nuisance, high-pitched buzzing, dengue fever, filariasis and malaria. The market survey in Bangladesh reveals that these substitutes include mosquito coils, mats, aerosols and vaporizers prepared with synthetic pyrethroid (SP) and organophosphate insecticides (Table 1). The literature reviews suggest that these substitutes contain along with the active ingredients (AI) other adjuvants and fillers, most of which have adverse effects and may cause several diseases in the affected human beings. Synthetic pyrethroids insecticides are heat stable and used in the treatment of mats, coils and vaporizers, e.g. allethrin and bioallethrin 4 %, *d*-allethrin 0.2 to 0.3 % *w*/*w*, *d*-*trans*-allethrin 0.1 to 0.15 % *w*/*w*, *s*-bioallethrin 1.9 %, etc. (Sharma 2001). On heating or burning of mats and liquids, these compounds vaporise without decomposition at temperatures up to 400 °C and produce repellent action on the mosquitoes. Breathing problems are the most common, and frequently this condition is accompanied with headache or eye irritation or both. Eye irritation is the next common complaint, and often it is accompanied with bronchial irritation, headache or skin reaction, cough, cold and running nose accompanied with fever or sneezing, wheezing and asthma, pain in the ear and throat as the reported adverse effects.

**Table 1:** Substitutes for DDT and their formulations in Bangladesh market and their hazard classifications

Sample #	Active ingredient/class^a^	Formulation type/product types^a^	WHO class	Health risks
01	Allethrin (SP)	Mat, Aerosol	III	a
02	*d*-allethrin (SP)	Mat, Coil	II	b
03	*d*-allethrin (Pynamine Fort) (SP)	Coil	II	b
04	*d*-*trans*-allethrin (SP)	Coil	II	b
05	Alpha Cypermethrin (SP)	WP, EC, SC	II	No evidence
06	Bioallethrin (SP)	Aerosol	II	a
07	S-Bio-allethrin (SP)	Coil	II	b
08	Chlorpyriphos (OP)	EC	II	No evidence
09	Cypermethrin (SP)	EC	II	No evidence
10	Deltamethrin (SP)	Chalk, EC, SC, WP, Flow, DP	II	No evidence
11	Diazinon (OP)	EC	II	No evidence
12	ETOC (Prallethrin) (SP)	Mat, Coil, Vaporizer	II	b
13	Fenthion (OP)	EC	II	No evidence
14	Fenitrothion (OP)	EC	II	No evidence
15	Imiprothin (SP)	Aerosol	III	a
16	Lambda Cyhalothrin (SP)	WP, EC	II	No evidence
17	Malathion (OP)	EC	III	No evidence
18	Metofluthrin (SP)	Coil		b
19	Permethrin (SP)	Aerosol, Powder, EC, WP, DP	II	No evidence
20	Phenthoate (OP)	Liquid, EC	II	No evidence
21	Pirimiphos Methyl (OP)	EC	III	No evidence
22	Pynamine Fort (SP)	Liquid vaporizer	III	a
23	Prallethrin (SP)	Aerosol, Coil, Mat, Vaporizer	II	b
24	Propoxur (Car)	Aerosol	II	a
25	Sumione (SP)	Coil	II	b
26	Temephos (OP)	EC, G	U	No evidence
27	Tetramethrin (SP)	RS, Aerosol	U	a
28	D-Tetramethrin (SP)	Aerosol	U	a
29	Transfluthrin (SP)	Coil	U	b

WHO class II means moderately hazardous (non-carcinogenic, non-teratogenic, etc.). WHO class III means slightly hazardous. WHO class U means unclassified. “No evidence” means literatures searched did not indicate significant health hazards under proper use. Letter “a” means health hazards are probable through high concentrations and long exposure due to organic solvents and propellant other than butane. Letter “b” means health hazards are most probable through emissions due to ingredients other than active ingredients

*SP* synthetic pyrethroid, *OP* organophosphate, *Car* carbamate

^a^Sample market survey and registry record

#### Probable health risks of mosquito coils/mats

SPs are major AI accounting for about 0.3–0.4 % of coil mass (Lukwa and Chandiwana 1998); the lowest lethal oral dose of which is 750 mg/kg for children and 1,000 mg/kg for adults (OHS 1987). Pyrethrins are of low chronic toxicity to humans and low reproductive toxicity in animals, although headache, nausea and dizziness are observed in male sprayers. The remaining components of mosquito coil are organic fillers, binders, dyes and other additives (Weili et al. 2003). Their combustion generates large amounts of submicrometer particles [(particulate matter <2.5 μm in diameter; P[M.sub.2.5])] and gaseous pollutants. Burning one mosquito coil generates P[M.sub.2.5] = burning 75–137 cigarettes. Coil smoke contains a suite of volatile organic compounds (VOCs), including human carcinogens and suspected carcinogens. Submicrometer particles can reach lower respiratory tract and may be coated with a wide range of organic compounds; some of which are carcinogens or suspected carcinogens (polycyclic aromatic hydrocarbons). Gas phase of coil smoke contains some carbonyl compounds (e.g. formaldehyde and acetaldehyde, as much as 55 %) with properties that can produce strong irritating effects on upper respiratory tract. The emission of formaldehyde from burning one coil is equal to burning 51 cigarettes. Long-term exposure to coil smoke induces asthma and persistent wheeze in children. Benz[*a*]anthracene, benzo[*a*]pyrene, benzo[*b*]fluoranthene, benzo[*k*]fluoranthene, dibenz[*a*,*h*] anthracene and indeno[1,2,3-ca]pyrene are classified by the US EPA as probable human carcinogens (EPA 1994). Acrolein, glyoxal and methyl-glyoxal known for their high reactivity, strong irritation effects and suspected carcinogenic effects are emitted from coil burning. Several VOCs including benzene, toluene, ethylbenzene, *p*,*m*,*o*-xylene and styrene are identified in coil smoke. All are known to cause adverse health effects. Mosquito coils from Asia and South America were reported to generate smoke from heating (or burning) containing submicron particles (<1 μm) coated with considerable amount of heavy metals, allethrin and a wide range of vapours such as phenol *o*-cresol, and allethrin used in the mats increased blood–brain barrier (BBB) permeability, suggesting a delayed maturity of BBB (Liu et al. 1987). When mosquito coils containing S-2 (octachlorodipropyl ether) are burned, they release a potent lung carcinogen called bis(chloromethyl) ether (Krieger et al. 2003).

#### Probable health risks of aerosol

An aerosol spray often consists of very small droplets of solvent, propellant, and AI. Major AIs of aerosols are SPs. Pyrethrin and pyrethroid illnesses have been reported in many cases (Walters et al. 2009). Most industrial aerosols contain organic solvents, which give off vapours that are dangerous, if breathed for too long or in too high concentrations. Excessive intake can cause headache, giddiness, mental confusion, blurred vision, nausea, weakness and fatigue, numbness of limbs and, in extreme cases, loss of consciousness (GPMU 2008; Harry et al. 1973; ATSDR 2003). Solvents in contact with skin cause irritation and defatting of the skin. Long-term effects of solvent exposure include damage to the heart, liver, kidneys and central nervous system. The most common propellant, butane, is of relatively low toxicity but is extremely flammable. But others are potentially hazardous in addition to flammability. Some of very small droplets of solvent, propellant and AI are ideally suited for breathing deep into lungs. Larger droplets are trapped in the nose, throat and upper part of the lungs.

#### Probable health risks of vaporiser

AIs of most vaporisers are SPs. By applying the electro-vaporizer “Nexa Lotte” plug-in mosquito killer, concentrations for *d*-allethrin were in the range of 5–12 and 0.5–2 μg/m^3^ for PBO, while with the “Paral” plug-in mosquito killer, concentrations of 0.4–5 μg/m^3^ for pyrethrins and 1–7 μg/m^3^ for PBO were measured (Berger-Preiss et al. 2009). Human biomonitoring data revealed urine concentrations of the metabolite (*E*)-*trans*-chrysanthemum dicarboxylic acid ((*E*)-*trans*-CDCA) between 1.7 and 7.1 μg/l after 5 min of exposure to the different sprays. Also, the use of electro-vaporizers led to (*E*)-*trans*-CDCA concentrations in the urine in the range of 1.0 to 6.2 μg/l (1–3 h exposure period).

### Alternative safe way of mosquito management

In the above context, the wise alternative to DDT for mosquito/malaria control may only refer to adoption of the integrated vector (mosquito) management (IVM) (William and Julie 1998; Sharma 2001; WHO 2004). IVM entails the use of a range of interventions of proven efficacy, separately or in combination, in order to implement more cost-effective control and reduce reliance on any single intervention. This strategy also serves to extend the useful life of insecticides and drugs by reducing the selection pressure for resistance development (WHO 2004). To prevent breeding of mosquitoes, IVM may include removal/cleaning of all breeding sites, e.g. bird baths, rain barrels, old automobile tires, ditches, unused swimming/water pools, tree holes, flower pots and roof gutters to avoid stagnant water/breeding of mosquitoes. To control larvae, the most logical approach includes examining each week the presence of larvae, using one or more of the insecticides such as *Bacillus thuringiensis* Berliner var. *israelensis*, methoprene—an insect growth regulator (Altosid or any available formulation) to second, third and fourth instars larvae in water, petroleum distillate oil and temephos. To control adults, the use of adulticides such as ultra-low volume application (malathion/chlorpyrifos/chlorpyrifos + permethrin), thermal fogging with malathion/chlorpyrifos/chlorpyrifos + permethrin as a space treatment against adult mosquitoes at night or early morning when the air is calm (less than 5 mph) or applying insecticide residual spray as barrier treatments to tall grasses, weeds, shrubs, fences and other harborages surrounding parks, playgrounds and residences to help reduce adult mosquito populations, and in endemic areas following WHO recommendation (CDC 2011) to avoid contact of adult mosquitoes using LLINs/ITNs.

## Conclusions and recommendation

From the above findings, it is concluded that any sole chemical substitute for DDT is health hazardous and the better alternative comprise the use of IVM package comprising microbial, physical, habitat sanitation and safer chemical methods including use of LLINs/ITNs. Therefore, wise recommendations to manage mosquitoes to prevent malaria should be adopted to replace DDT with IVM and not by any other single method, which may cause health and environmental hazards as much as caused by DDT.
